# Supplementary material for: Comparative Studies on Resampling Techniques in Machine Learning and Deep Learning Models for Drug-Target Interaction Prediction
Source: Molecules. 2023 Feb 9;28(4):1663. doi: 10.3390/molecules28041663 (PMC9964614; doi:10.3390/molecules28041663)
Supplement: Supplementary file 1 [file molecules-28-01663-s001.zip › molecules-2010254-supplementary-revised.pdf]

molecules

MDPI

Supplementary Materials

Comparative Studies on Resampling Techniques in Machine Learning and Deep Learning Models for Drug-Target Interaction Prediction

Azwaar Khan Azlim Khan and Nurul Hashimah Ahamed Hassain Malim

BRAF achieved the highest accuracy of 94.51% when SMOTETomek was applied on the RF classifier, where an increase of 5.04% is noticed from the original accuracy value of 89.47% when no resampling method was applied (Refer to Figure S1). Furthermore, when no resampling method was applied, the precision value is the highest (99.93%), and a drop of 7.83% is noticed when ADASYN was used, while the other resampling methods showed a drop in precision values even larger than 7.83% (Refer to Figure S1). In terms of recall, BRAF performs the best when SVM-SMOTE was used with the GNB classifier, yielding a total recall value of 92.58%, which is a 3.72% increase from when no resampling method is used in GNB, with a recall value of 88.86% (Refer to Figure S1). In general, RF is the best classifier, outperforming all the other classifiers when paired with resampling methods and SVM and DT turns out to be the weakest classifiers in terms of accuracy, precision and recall.

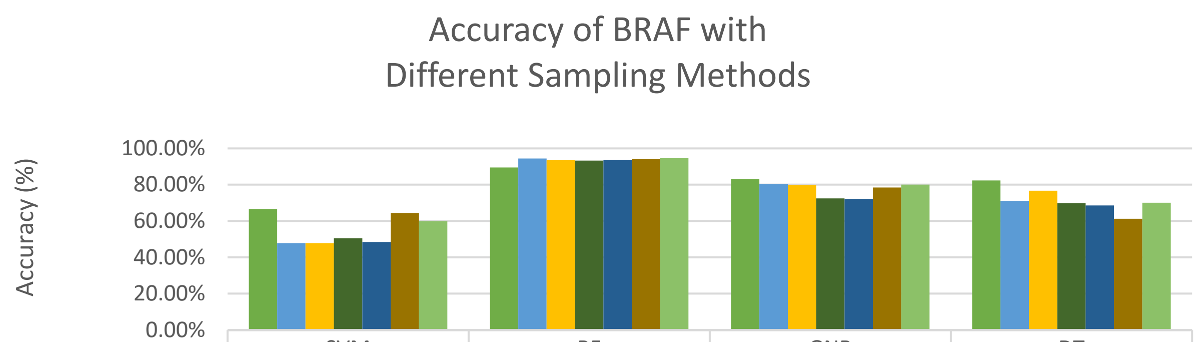

	SVM	RF	GNB	DT
None	66.57%	89.47%	82.99%	82.29%
SMOTE	47.83%	94.33%	80.31%	71.05%
RUS	47.82%	93.48%	79.80%	76.65%
ADASYN	50.50%	93.26%	72.40%	69.80%
BorderlineSMOTE	48.42%	93.47%	72.17%	68.53%
SVM-SMOTE	64.39%	93.98%	78.41%	61.18%
SMOTETomek	59.91%	94.51%	79.96%	70.05%

(a)

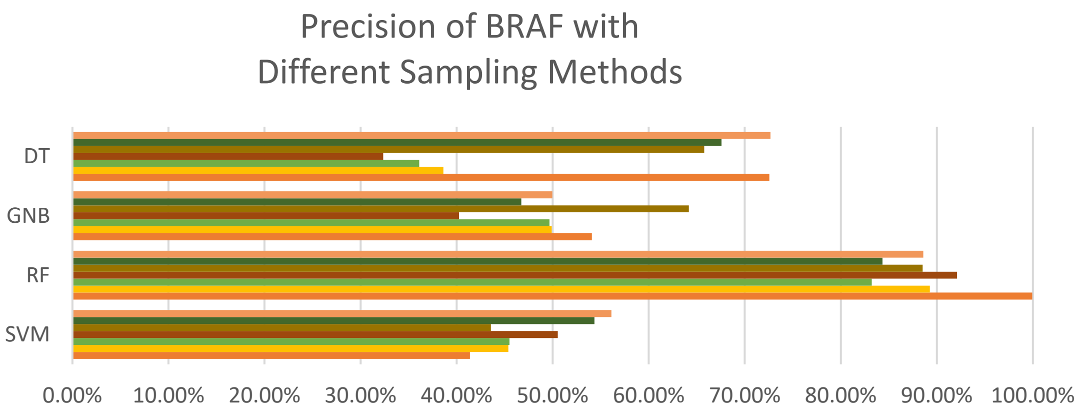

	SVM	RF	GNB	DT
SMOTETomek	56.11%	88.59%	49.97%	72.67%
SVM-SMOTE	54.35%	84.34%	46.74%	67.58%
BorderlineSMOTE	43.57%	88.52%	64.18%	65.78%
ADASYN	50.53%	92.10%	40.25%	32.35%
RUS	45.49%	83.23%	49.66%	36.11%
SMOTE	45.39%	89.28%	49.91%	38.61%
None	41.39%	99.93%	54.08%	72.57%

(b)

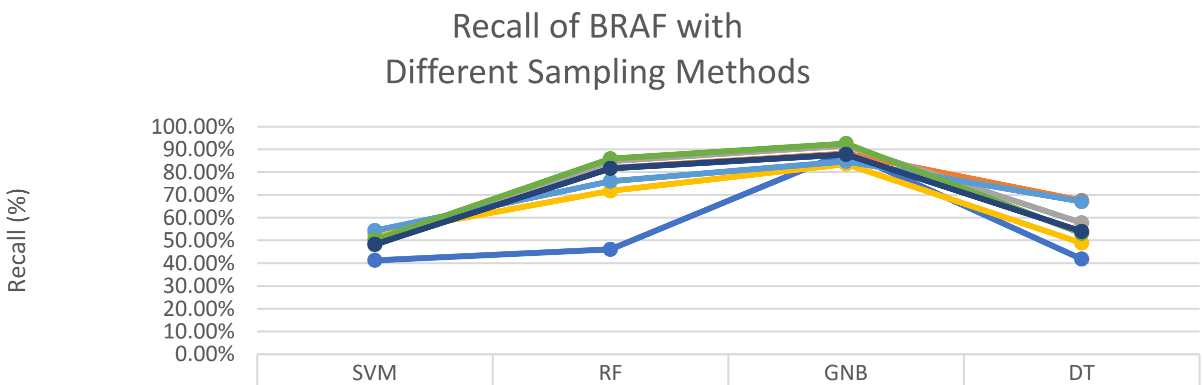

	SVM	RF	GNB	DT
None	41.26%	46.08%	88.86%	41.82%
SMOTE	48.55%	81.86%	88.21%	67.50%
RUS	47.97%	84.83%	91.63%	57.72%
ADASYN	52.79%	71.77%	83.56%	48.88%
BorderlineSMOTE	54.38%	76.00%	84.73%	67.06%
SVM-SMOTE	50.25%	85.90%	92.58%	53.08%
SMOTETomek	48.29%	81.63%	87.76%	53.96%

(c)

Figure S1. The accuracy, precision and recall values for BRAF. **(a)** Accuracy of BRAF when different sampling methods are applied; **(b)** Precision of BRAF when different sampling methods are applied; **(c)** Recall of BRAF when different sampling methods are applied.

For CDK-6, the accuracy is the highest when SVM-SMOTE is used with the RF classifier (99.70%), which a slight increase from when RF is applied with no resampling method (Refer to Figure S2). In terms of precision, RF is the best classifier when no

resampling method is applied, yielding a precision value of 99.82% and in terms of recall, it was found that GNB with SVM-SMOTE achieved the highest recall with a value of 96.56%, which is a 7.41% increase from the original value when GNB is applied with no resampling method (Refer to Figure S2). Similar to BRAF, SVM and DT are the weakest classifiers when paired with resampling methods in terms of accuracy, precision and recall while RF is the best classifier.

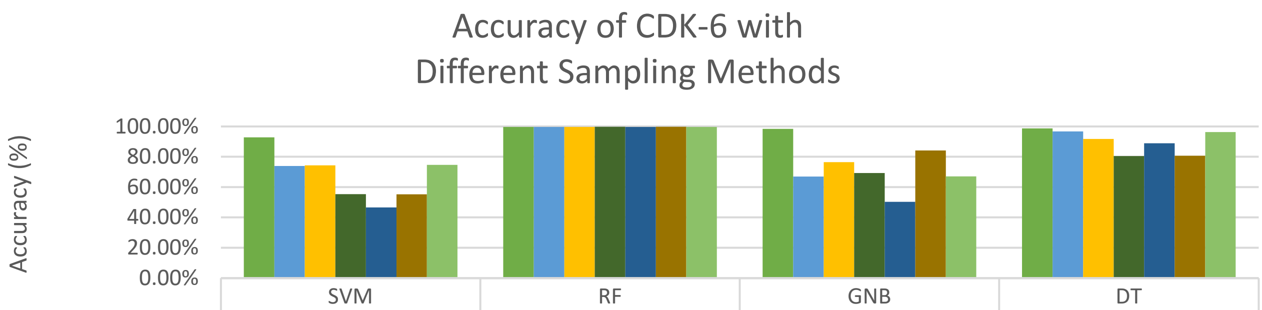

	SVM	RF	GNB	DT
None	92.79%	99.63%	98.33%	98.63%
SMOTE	73.93%	99.66%	66.94%	96.67%
RUS	74.29%	99.60%	76.42%	91.73%
ADASYN	55.35%	99.69%	69.28%	80.40%
BorderlineSMOTE	46.52%	99.68%	50.22%	88.85%
SVM-SMOTE	55.24%	99.70%	84.13%	80.60%
SMOTETomek	74.62%	99.65%	66.98%	96.28%

(a)

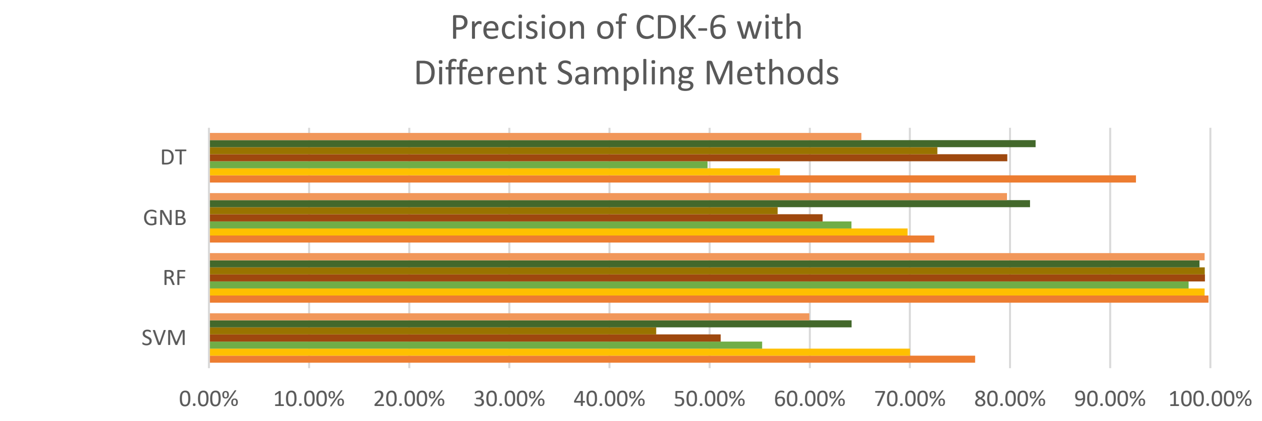

	SVM	RF	GNB	DT
SMOTETomek	59.96%	99.43%	79.70%	65.15%
SVM-SMOTE	64.17%	98.92%	81.99%	82.56%
BorderlineSMOTE	44.66%	99.44%	56.79%	72.74%
ADASYN	51.11%	99.46%	61.29%	79.72%
RUS	55.25%	97.84%	64.16%	49.80%
SMOTE	70.01%	99.44%	69.77%	57.02%
None	76.51%	99.82%	72.45%	92.57%

(b)

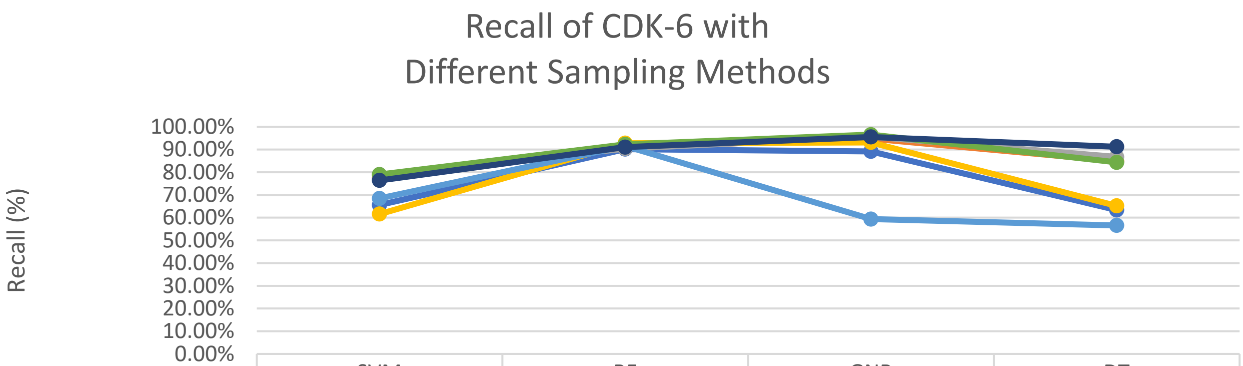

	SVM	RF	GNB	DT
None	65.58%	90.19%	89.15%	63.36%
SMOTE	77.28%	91.05%	94.84%	84.85%
RUS	78.99%	90.36%	95.70%	86.74%
ADASYN	61.61%	92.77%	93.12%	65.18%
BorderlineSMOTE	68.47%	91.56%	59.35%	56.58%
SVM-SMOTE	78.99%	92.25%	96.56%	84.33%
SMOTETomek	76.43%	91.05%	95.52%	91.23%

(c)

Figure S2. The accuracy, precision and recall values for CDK-6. **(a)** Accuracy of CDK-6 when different sampling methods are applied; **(b)** Precision of CDK-6 when different sampling methods are applied; **(c)** Recall of CDK-6 when different sampling methods are applied.

Next, in HER2, RF is also still the best classifier when paired with various resampling methods. The accuracy is the highest when SMOTETomek was applied with the RF classifier (94.39%), which is a 2.36% increase from when no resampling is applied to RF and in terms of precision, it is surprising that RF with no resampling method is the highest (96.61%) while a huge drop in the precision value is observed when resampling methods are applied (Refer to Figure S3). It was also found that when SVM-SMOTE is applied with the GNB classifier, the recall is the highest, which is a value of 90.89% (Refer to Figure S3).

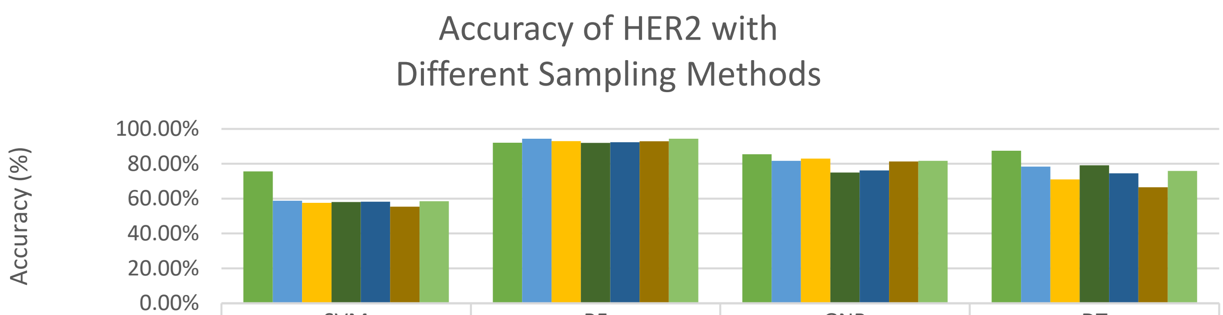

	SVM	RF	GNB	DT
None	75.58%	92.03%	85.43%	87.46%
SMOTE	58.79%	94.32%	81.66%	78.35%
RUS	57.56%	92.97%	82.96%	71.02%
ADASYN	58.08%	91.98%	74.99%	79.11%
BorderlineSMOTE	58.20%	92.36%	76.16%	74.49%
SVM-SMOTE	55.37%	92.92%	81.27%	66.51%
SMOTETomek	58.51%	94.39%	81.68%	75.88%

(a)

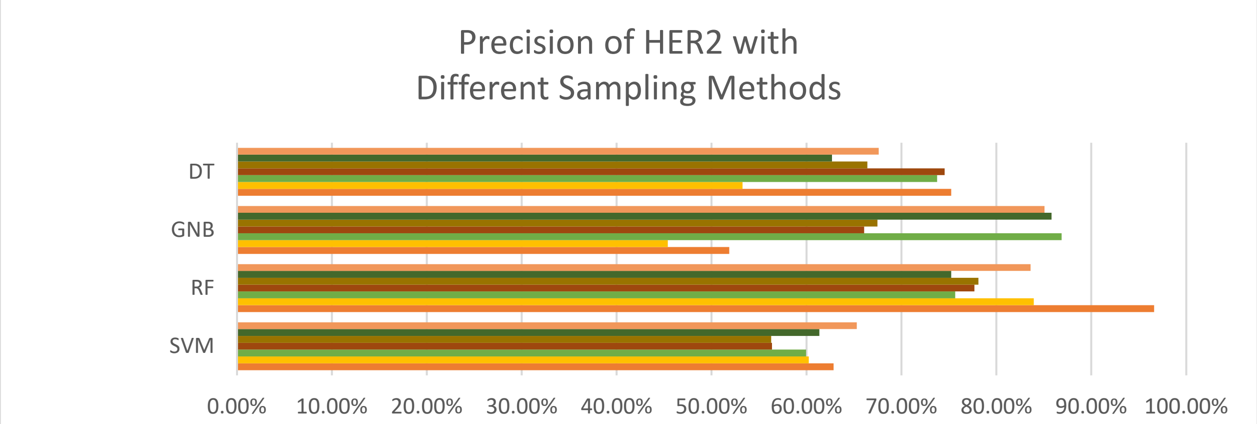

	SVM	RF	GNB	DT
SMOTETomek	65.29%	83.61%	85.07%	67.61%
SVM-SMOTE	61.36%	75.25%	85.82%	62.69%
BorderlineSMOTE	56.28%	78.11%	67.47%	66.42%
ADASYN	56.37%	77.70%	66.08%	74.56%
RUS	59.96%	75.67%	86.88%	73.76%
SMOTE	60.24%	83.95%	45.40%	53.27%
None	62.85%	96.61%	51.86%	75.25%

(b)

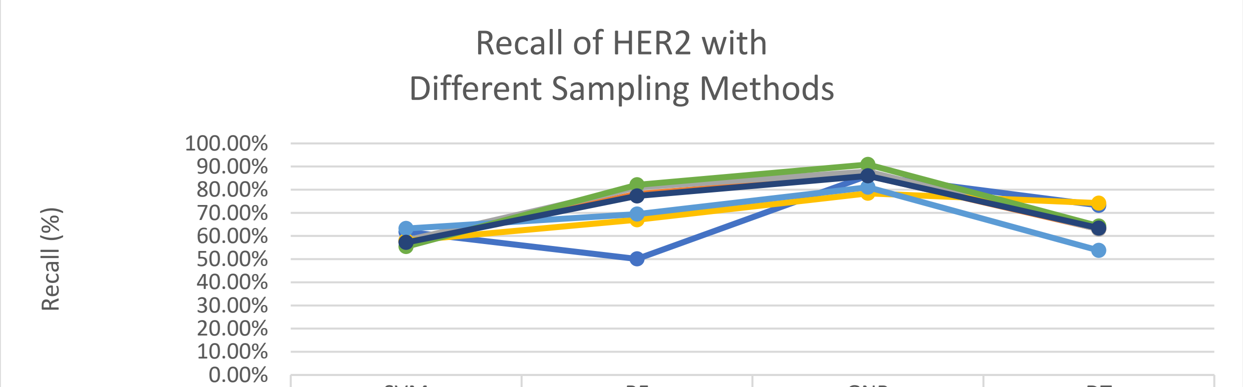

	SVM	RF	GNB	DT
None	61.82%	50.08%	86.11%	73.20%
SMOTE	57.50%	78.11%	86.07%	63.00%
RUS	58.12%	80.95%	87.84%	62.96%
ADASYN	57.91%	66.98%	78.44%	74.17%
BorderlineSMOTE	63.24%	69.46%	81.08%	53.75%
SVM-SMOTE	55.44%	82.07%	90.89%	64.25%
SMOTETomek	57.26%	77.29%	85.94%	63.39%

(c)

Figure S3. The accuracy, precision and recall values for HER2. **(a)** Accuracy of HER2 when different sampling methods are applied; **(b)** Precision of HER2 when different sampling methods are applied; **(c)** Recall of HER2 when different sampling methods are applied.

For KRAS, the accuracy is the highest when both SVM-SMOTE and SMOTETomek were applied on the RF classifier, with both achieving an accuracy of 99.94%, which is a

slight increase from when no resampling is applied on RF with a value of 99.82% (Refer to Figure S4). PD-1 also observed a high accuracy value of 99.96% when ADASYN, SVM-SMOTE and SMOTETomek were applied on the RF classifier (Refer to Figure S6). The precision of KRAS and PD-1 achieved an outstanding 100% when SMOTE, ADASYN, BorderlineSMOTE and SMOTETomek is applied (Refer to Figure S4 and Figure S6). The precision of PD-1 also reached 100% when SVM-SMOTE was used with the RF classifier. It is also very interesting to note that the precision for KRAS and PD-1 also reached 100% when no resampling method was applied on the RF classifier (Refer to Figures S4 and S6). Furthermore, the recall for both KRAS and PD-1 was also the highest when GNB was paired with SMOTE for KRAS, achieving a recall of 99.50%, and 99.36% for PD-1 when GNB was paired with SVM-SMOTE (Refer to Figures S4 and S6). Both of these recall values showed an increase from the original value when no sampling was applied, whereby a 7.92% increase was seen for KRAS and a 1.91% increase for PD-1 (Refer to Figures S4 and S6).

Then, for the MEK1 activity class, the highest accuracy is 99.95% when ADASYN and SMOTETomek was paired with the RF classifier (Refer to Figure S5). In terms of precision, the highest value is achieved when ADAYSN was paired with the RF classifier, which is a value of 97.50% (Refer to Figure S5). The same precision value was observed when no resampling method was applied on the RF classifier as well. Finally, the highest recall value is also 97.50% and this value is achieved when the GNB classifier is applied with SMOTE, BorderlineSMOTE and SMOTETomek (Refer to Figure 5S).

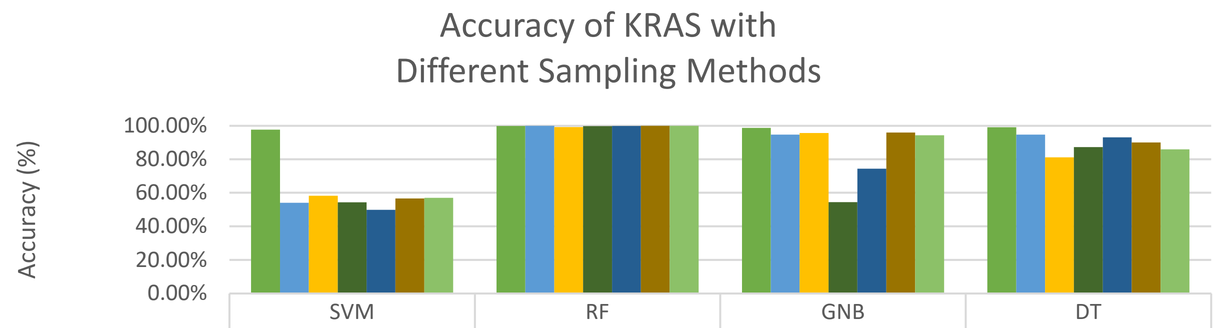

	SVM	RF	GNB	DT
None	97.65%	99.82%	98.74%	99.11%
SMOTE	54.04%	99.93%	94.71%	94.65%
RUS	58.27%	99.21%	95.63%	81.16%
ADASYN	54.36%	99.76%	54.43%	87.22%
BorderlineSMOTE	49.82%	99.83%	74.34%	93.07%
SVM-SMOTE	56.66%	99.94%	95.93%	89.99%
SMOTETomek	57.04%	99.94%	94.36%	85.88%

(a)

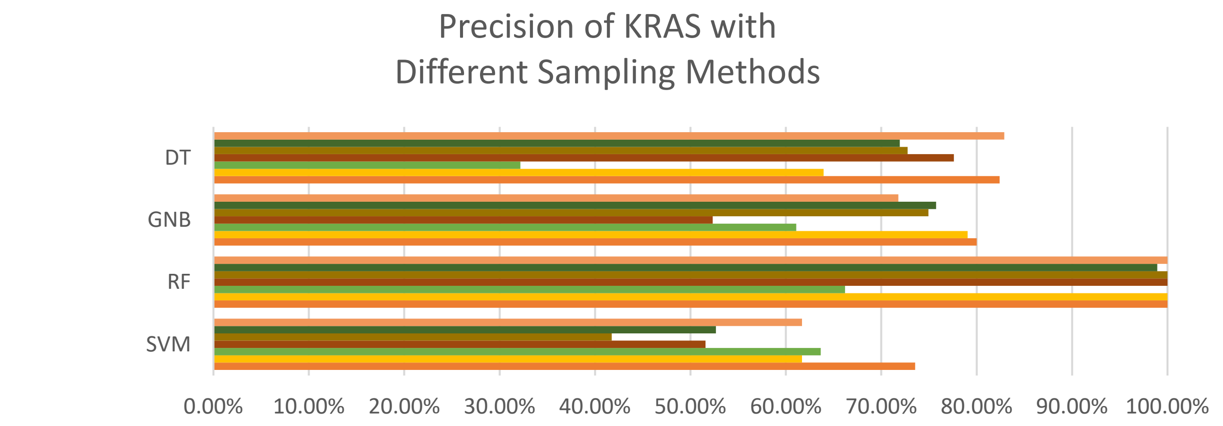

	SVM	RF	GNB	DT
SMOTETomek	61.70%	100.00%	71.78%	82.89%
SVM-SMOTE	52.66%	98.93%	75.75%	71.94%
BorderlineSMOTE	41.76%	100.00%	74.95%	72.76%
ADASYN	51.58%	100.00%	52.33%	77.62%
RUS	63.66%	66.20%	61.09%	32.18%
SMOTE	61.69%	100.00%	79.06%	63.96%
None	73.56%	100.00%	80.01%	82.40%

(b)

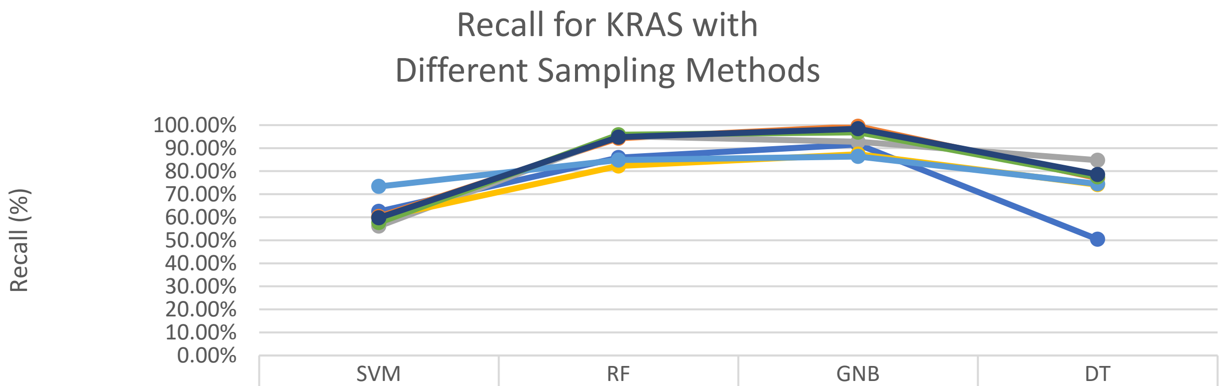

	SVM	RF	GNB	DT
None	62.53%	85.90%	91.58%	50.42%
SMOTE	60.29%	94.26%	99.50%	76.97%
RUS	56.16%	95.32%	92.74%	84.76%
ADASYN	59.71%	82.24%	87.40%	74.13%
BorderlineSMOTE	73.37%	84.83%	86.38%	74.48%
SVM-SMOTE	57.65%	95.80%	96.87%	77.46%
SMOTETomek	59.74%	94.78%	98.42%	78.56%

(c)

Figure S4. The accuracy, precision and recall values for KRAS. **(a)** Accuracy of KRAS when different sampling methods are applied; **(b)** Precision of KRAS when different sampling methods are applied; **(c)** Recall of KRAS when different sampling methods are applied.

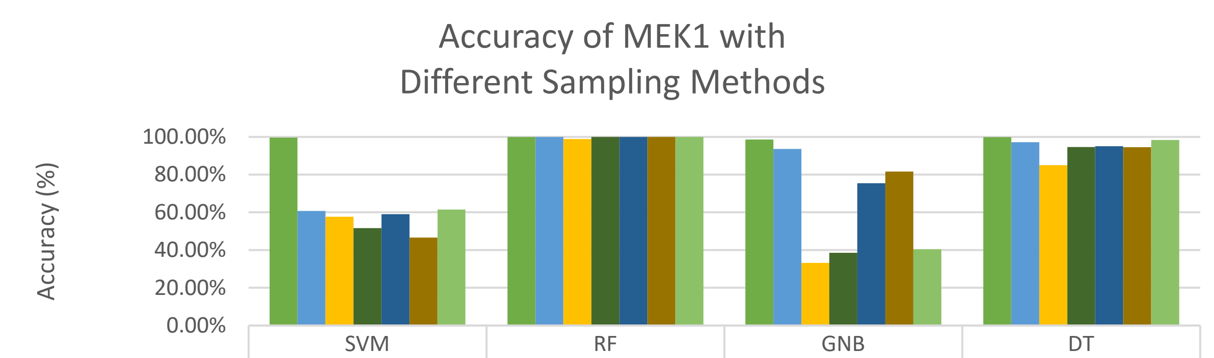

	SVM	RF	GNB	DT
None	99.59%	99.93%	98.54%	99.81%
SMOTE	60.74%	99.94%	93.58%	97.17%
RUS	57.65%	98.82%	33.23%	84.99%
ADASYN	51.59%	99.95%	38.53%	94.62%
BorderlineSMOTE	59.01%	99.94%	75.35%	95.04%
SVM-SMOTE	46.58%	99.94%	81.56%	94.54%
SMOTETomek	61.46%	99.95%	40.41%	98.32%

(a)

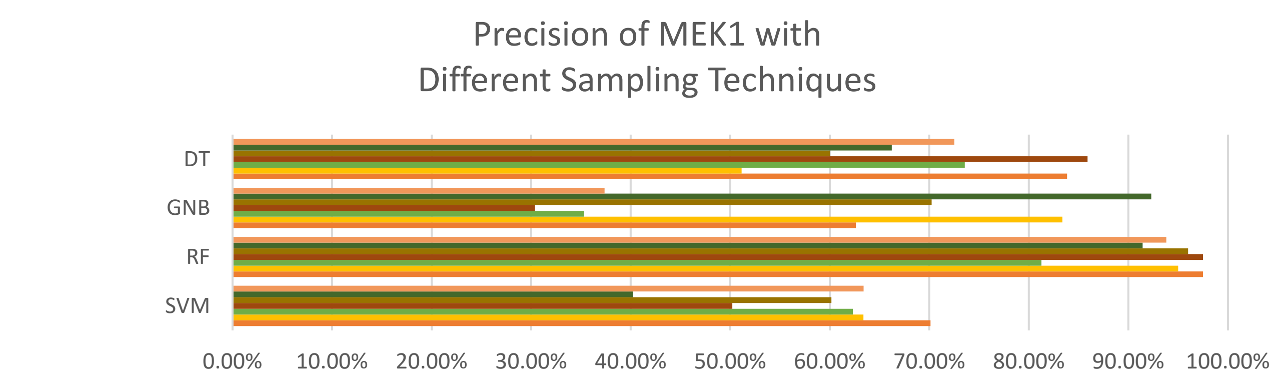

	SVM	RF	GNB	DT
SMOTETomek	63.39%	93.81%	37.39%	72.53%
SVM-SMOTE	40.20%	91.43%	92.30%	66.23%
BorderlineSMOTE	60.17%	96.00%	70.24%	60.00%
ADASYN	50.21%	97.50%	30.38%	85.90%
RUS	62.33%	81.27%	35.31%	73.57%
SMOTE	63.39%	95.00%	83.37%	51.14%
None	70.12%	97.50%	62.63%	83.83%

(b)

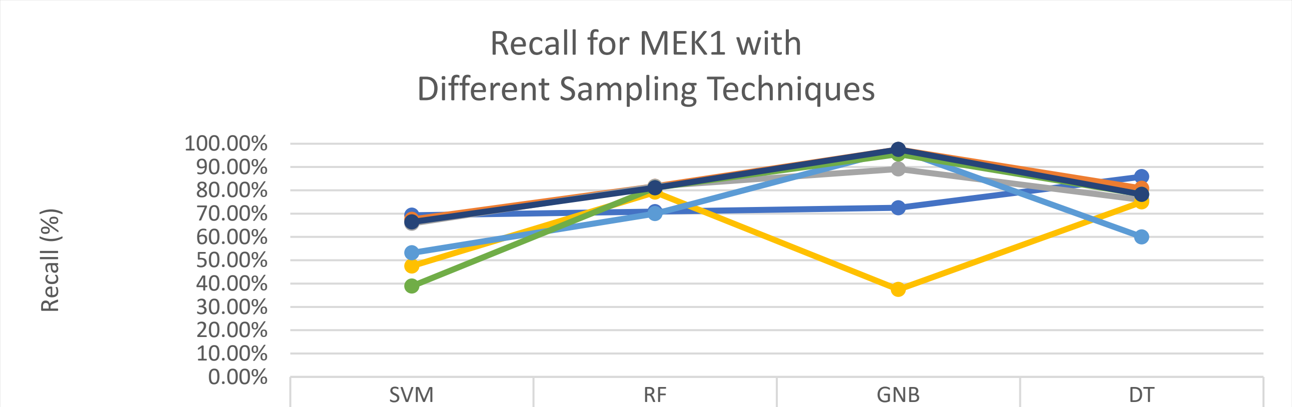

	SVM	RF	GNB	DT
None	69.31%	70.83%	72.50%	85.83%
SMOTE	67.50%	81.67%	97.50%	80.83%
RUS	65.83%	81.67%	89.17%	75.83%
ADASYN	47.50%	79.17%	37.50%	75.00%
BorderlineSMOTE	53.21%	70.00%	97.50%	60.00%
SVM-SMOTE	38.93%	81.07%	95.50%	78.25%
SMOTETomek	66.43%	81.07%	97.50%	78.21%

(c)

Figure S5. The accuracy, precision and recall values for MEK1. **(a)** Accuracy of MEK1 when different sampling methods are applied; **(b)** Precision of MEK1 when different sampling methods are applied; **(c)** Recall of MEK1 when different sampling methods are applied.

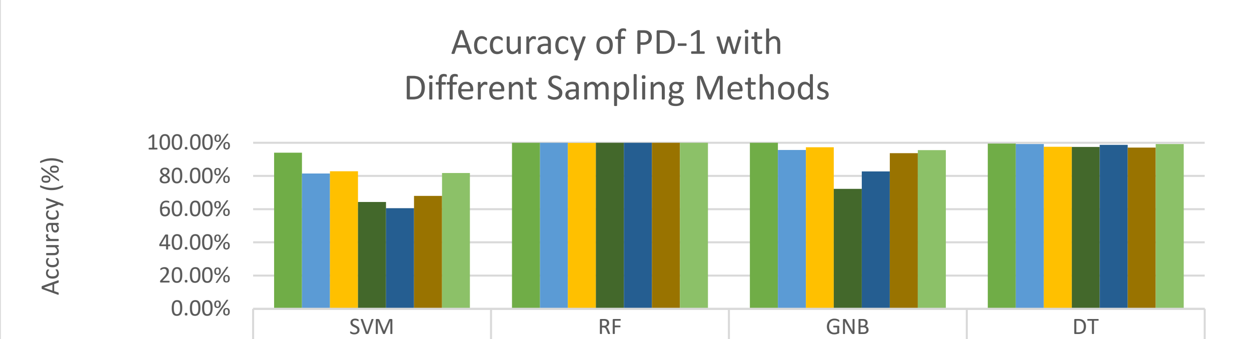

	SVM	RF	GNB	DT
None	94.00%	99.95%	99.92%	99.47%
SMOTE	81.50%	99.95%	95.64%	99.23%
RUS	82.81%	99.90%	97.29%	97.60%
ADASYN	64.33%	99.96%	72.22%	97.44%
BorderlineSMOTE	60.53%	99.95%	82.69%	98.67%
SVM-SMOTE	67.97%	99.96%	93.71%	97.06%
SMOTETomek	81.80%	99.96%	95.55%	99.19%

(a)

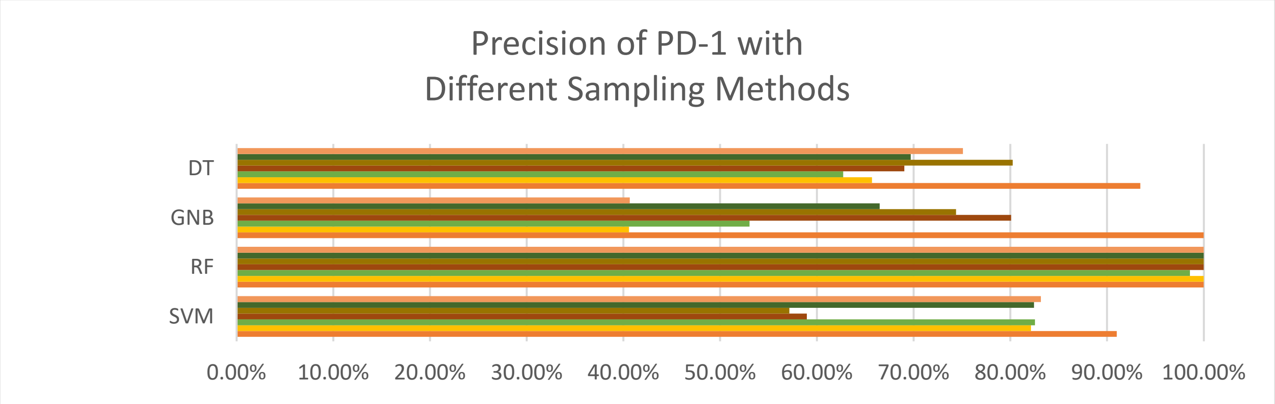

	SVM	RF	GNB	DT
SMOTETomek	83.16%	100.00%	40.65%	75.10%
SVM-SMOTE	82.45%	100.00%	66.50%	69.71%
BorderlineSMOTE	57.16%	100.00%	74.39%	80.25%
ADASYN	58.96%	100.00%	80.08%	69.04%
RUS	82.54%	98.57%	53.04%	62.71%
SMOTE	82.13%	100.00%	40.58%	65.71%
None	91.00%	100.00%	100.00%	93.44%

(b)

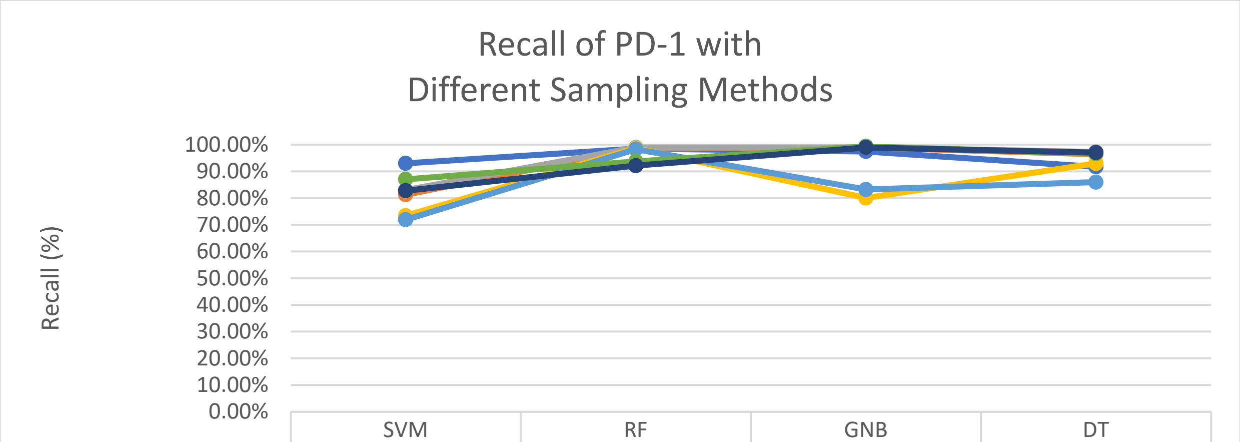

	SVM	RF	GNB	DT
None	93.00%	98.51%	97.45%	91.70%
SMOTE	81.28%	98.51%	98.94%	96.38%
RUS	82.98%	98.94%	98.94%	97.23%
ADASYN	73.40%	98.72%	80.00%	92.98%
BorderlineSMOTE	71.92%	98.30%	83.19%	85.96%
SVM-SMOTE	87.02%	93.72%	99.36%	96.60%
SMOTETomek	82.77%	92.12%	98.94%	97.02%

(c)

Figure S6. The accuracy, precision and recall values for PD-1. **(a)** Accuracy of PD-1 when different sampling methods are applied; **(b)** Precision of PD-1 when different sampling methods are applied; **(c)** Recall of PD-1 when different sampling methods are applied.

Next, for PDGFR-A, it was found that when SMOTE is applied on the RF classifier, the accuracy is the highest, yielding an accuracy value of 97.38% (Refer to Figure S7). However, in terms of precision, PDGFR-A seems to be performing well when no

resampling method is used on the RF classifier, achieving a value of 96.54% (Refer to Figure S7). Nevertheless, a drop in the precision value can be seen across most of the classifiers when different resampling methods are applied, except for GNB in which when SMOTE is applied on the GNB classifier, a sharp increase of 24.75% in the precision value can be seen (Refer to Figure S7). For the recall of PDGFR-A, the value is the highest when BorderlineSMOTE is applied with the RF classifier with a value of 88.88% (Refer to Figure S7). Now for PDGFR-B, the accuracy value is the highest when the RF classifier is applied with SMOTE and BorderlineSMOTE, achieving a value of 99.87% respectively (Refer to Figure S8). The highest precision value recorded was 92.46%, and this was when RUS was paired with the RF classifier. However, when RUS is applied to the other classifiers, a sharp decrease is observed in the precision value (Refer to Figure S8). In terms of recall, the highest value achieved is when RUS was paired the RF classifier (99.50%) while a drop in recall values can also be seen in both the SVM and GNB classifiers when different resampling methods are applied, especially with ADASYN, BorderlineSMOTE and SVM-SMOTE, which is a similar case to the nature of the precision values of PDGFR-B mentioned earlier (Refer to Figure S8).

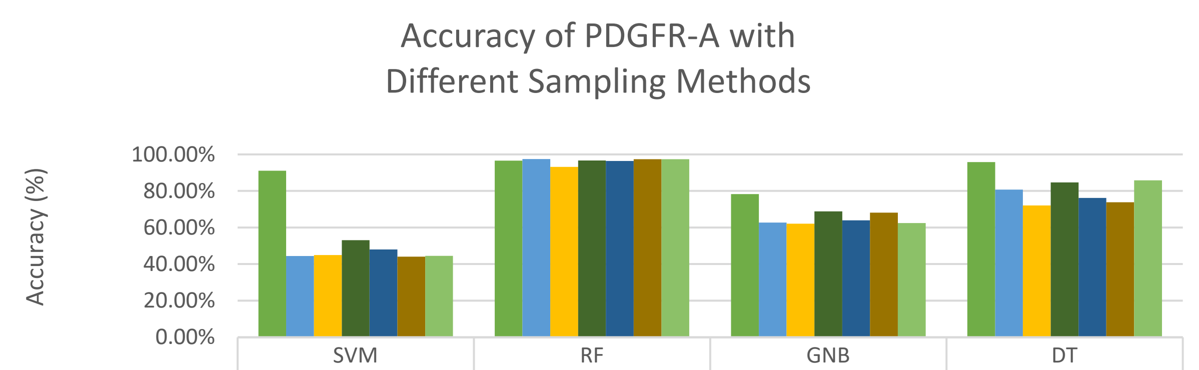

	SVM	RF	GNB	DT
None	91.01%	96.56%	78.25%	95.76%
SMOTE	44.40%	97.38%	62.69%	80.69%
RUS	44.88%	93.15%	62.03%	72.06%
ADASYN	53.05%	96.58%	68.79%	84.60%
BorderlineSMOTE	48.00%	96.33%	63.94%	76.12%
SVM-SMOTE	44.04%	97.32%	68.06%	73.76%
SMOTETomek	44.50%	97.34%	62.41%	85.73%

(a)

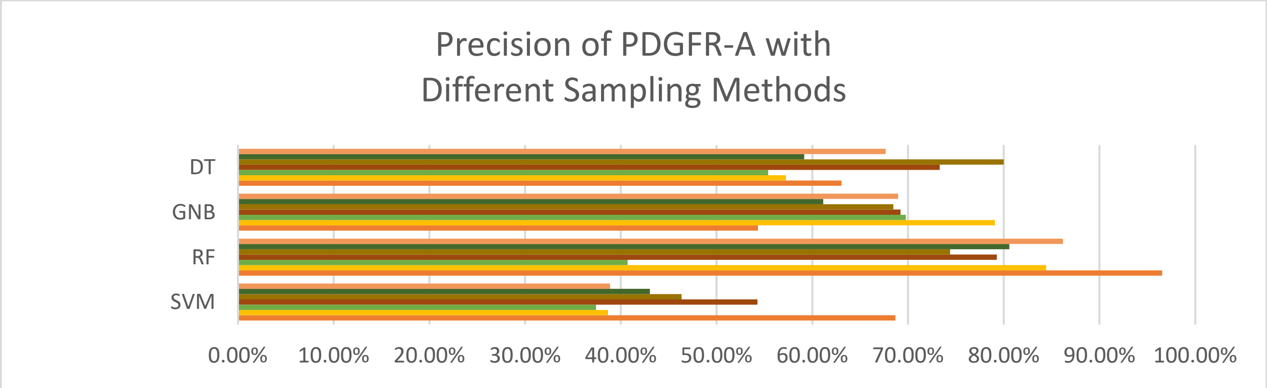

	SVM	RF	GNB	DT
SMOTETomek	38.88%	86.19%	68.96%	67.66%
SVM-SMOTE	43.04%	80.58%	61.14%	59.15%
BorderlineSMOTE	46.34%	74.40%	68.47%	80.00%
ADASYN	54.27%	79.29%	69.22%	73.31%
RUS	37.40%	40.71%	69.76%	55.40%
SMOTE	38.66%	84.44%	79.07%	57.25%
None	68.69%	96.54%	54.32%	63.04%

(b)

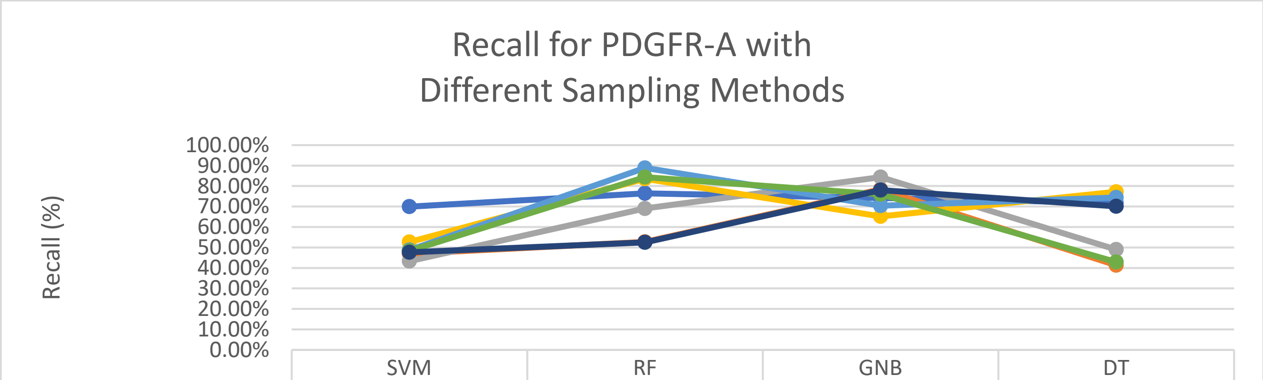

	SVM	RF	GNB	DT
None	69.99%	76.51%	74.16%	71.76%
SMOTE	46.94%	52.76%	78.44%	41.31%
RUS	43.36%	69.06%	84.39%	48.98%
ADASYN	52.67%	83.57%	65.18%	77.22%
BorderlineSMOTE	48.76%	88.88%	70.45%	74.43%
SVM-SMOTE	48.34%	84.42%	75.97%	42.87%
SMOTETomek	47.65%	52.49%	78.04%	70.10%

(c)

Figure S7. The accuracy, precision and recall values for PDGFR-A. **(a)** Accuracy of PDGFR-A when different sampling methods are applied; **(b)** Precision of PDGFR-A when different sampling methods are applied; **(c)** Recall of PDGFR-A when different sampling methods are applied. 96 97 98

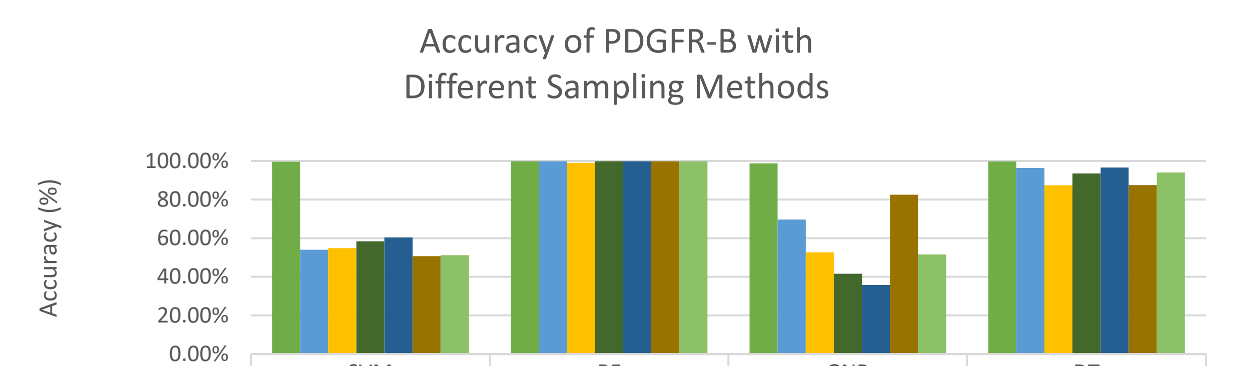

	SVM	RF	GNB	DT
None	99.60%	99.85%	98.77%	99.71%
SMOTE	54.05%	99.87%	69.65%	96.36%
RUS	54.87%	98.99%	52.66%	87.39%
ADASYN	58.42%	99.85%	41.57%	93.50%
BorderlineSMOTE	60.41%	99.87%	35.82%	96.57%
SVM-SMOTE	50.70%	99.86%	82.48%	87.46%
SMOTETomek	51.21%	99.84%	51.61%	94.00%

(a)

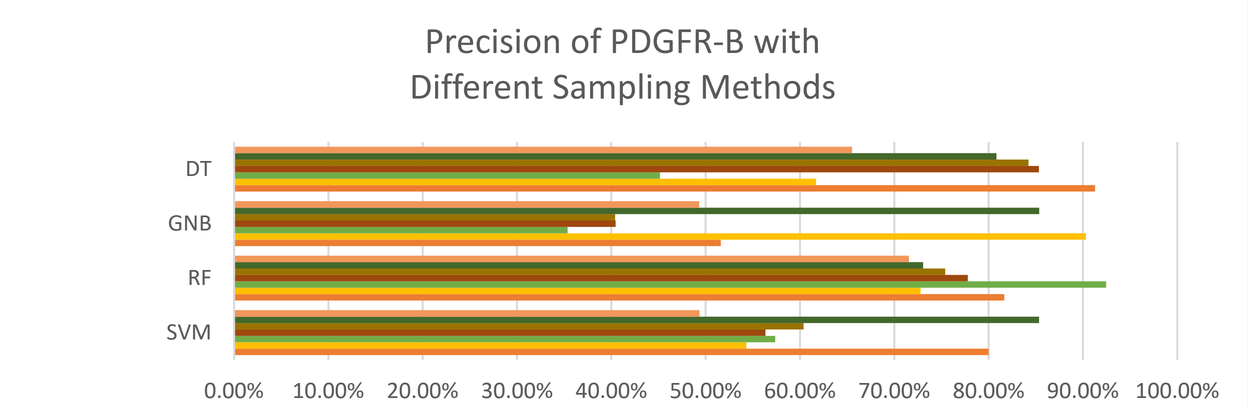

	SVM	RF	GNB	DT
SMOTETomek	49.34%	71.54%	49.31%	65.52%
SVM-SMOTE	85.35%	73.06%	85.36%	80.84%
BorderlineSMOTE	60.37%	75.40%	40.41%	84.22%
ADASYN	56.34%	77.79%	40.45%	85.32%
RUS	57.38%	92.46%	35.36%	45.15%
SMOTE	54.31%	72.79%	90.31%	61.70%
None	80.00%	81.67%	51.60%	91.29%

(b)

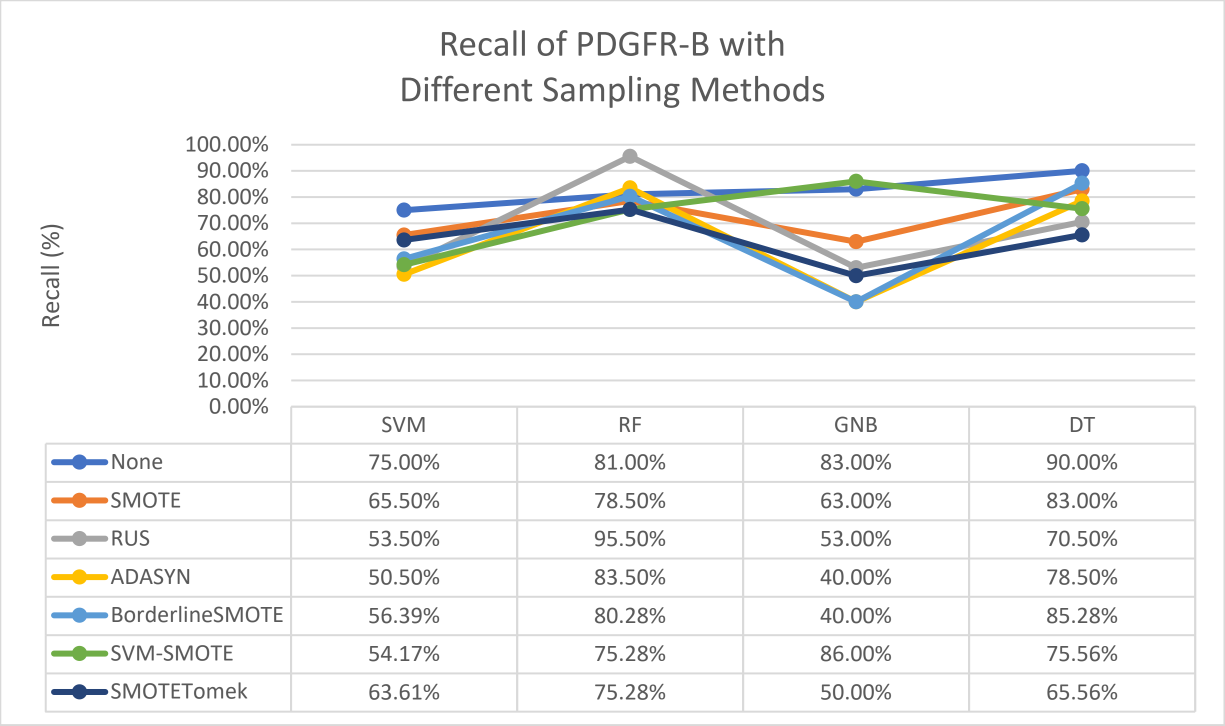

	SVM	RF	GNB	DT
None	75.00%	81.00%	83.00%	90.00%
SMOTE	65.50%	78.50%	63.00%	83.00%
RUS	53.50%	95.50%	53.00%	70.50%
ADASYN	50.50%	83.50%	40.00%	78.50%
BorderlineSMOTE	56.39%	80.28%	40.00%	85.28%
SVM-SMOTE	54.17%	75.28%	86.00%	75.56%
SMOTETomek	63.61%	75.28%	50.00%	65.56%

(c)

Figure S8. The accuracy, precision and recall values for PDGFR-B. **(a)** Accuracy of PDGFR-B when different sampling methods are applied; **(b)** Precision of PDGFR-B when different sampling methods are applied; **(c)** Recall of PDGFR-B when different sampling methods are applied.

Additionally, the accuracy for VEGFR-1 is the highest when SMOTETomek is applied on the RF classifier, achieving a value of 93.96%, which is a 1.04% increase compared to its original accuracy value when no resampling method is used with the RF classifier, 92.92% (Refer to Figure S9). In terms of precision, it is interesting to note that when no resampling method is applied on the RF classifier, the precision is the highest with a value of 98.55%, similar to activity classes KRAS and PD-1 (Refer to Figures S4, S6 and S9). Similarly, the same case is also happening to the recall value of VEGFR-1, whereby the value is the highest when no resampling method is used (similar to activity classes KRAS and PD-1), achieving a value of 88.94% (Refer to Figures S4, S6 and S9). Finally, the accuracy of VEGFR-2 is the highest when SVM-SMOTE is paired with the RF classifier, yielding an accuracy value of 91.94%, which is a 10.99% increase from the original value of 80.95% when no resampling was used (Refer to Figure S10). In addition, when RF is paired with BorderlineSMOTE, the best precision value recorded was 87.08% and in terms of recall, the value is surprisingly the highest when no resampling method is used on the RF classifier, yielding a value of 99.48% which is similar to the case of the recall values of the activity classes KRAS, PD-1 and VEGFR-1 (Refer to Figures S4, S6, S9 and S10).

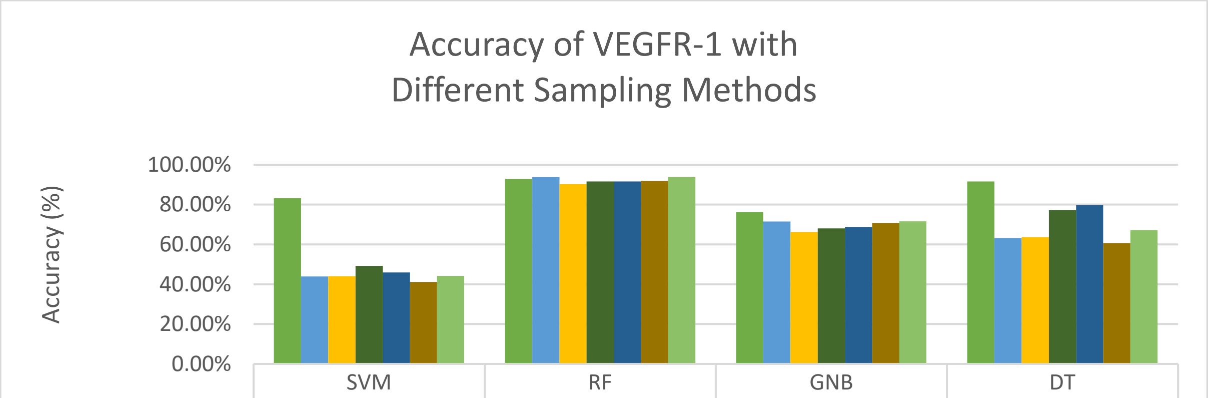

	SVM	RF	GNB	DT
None	83.14%	92.92%	76.18%	91.60%
SMOTE	43.92%	93.78%	71.51%	63.16%
RUS	44.02%	90.21%	66.37%	63.71%
ADASYN	49.22%	91.63%	68.05%	77.23%
BorderlineSMOTE	45.91%	91.61%	68.75%	79.84%
SVM-SMOTE	41.15%	91.94%	70.84%	60.65%
SMOTETomek	44.20%	93.96%	71.61%	67.16%

(a)

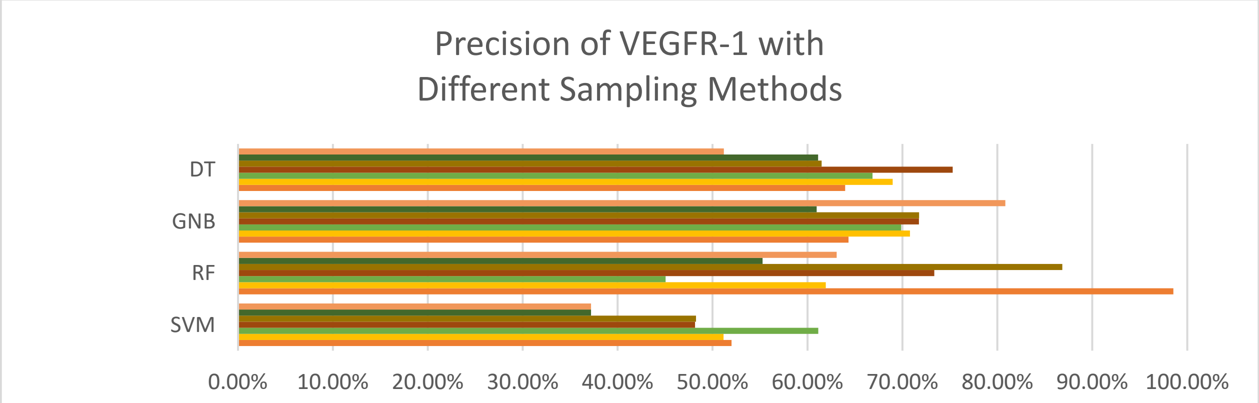

	SVM	RF	GNB	DT
SMOTETomek	37.20%	63.08%	80.84%	51.19%
SVM-SMOTE	37.19%	55.26%	60.97%	61.11%
BorderlineSMOTE	48.26%	86.84%	71.76%	61.49%
ADASYN	48.15%	73.36%	71.74%	75.30%
RUS	61.14%	45.06%	69.86%	66.85%
SMOTE	51.15%	61.93%	70.80%	68.98%
None	52.00%	98.55%	64.32%	63.96%

(b)

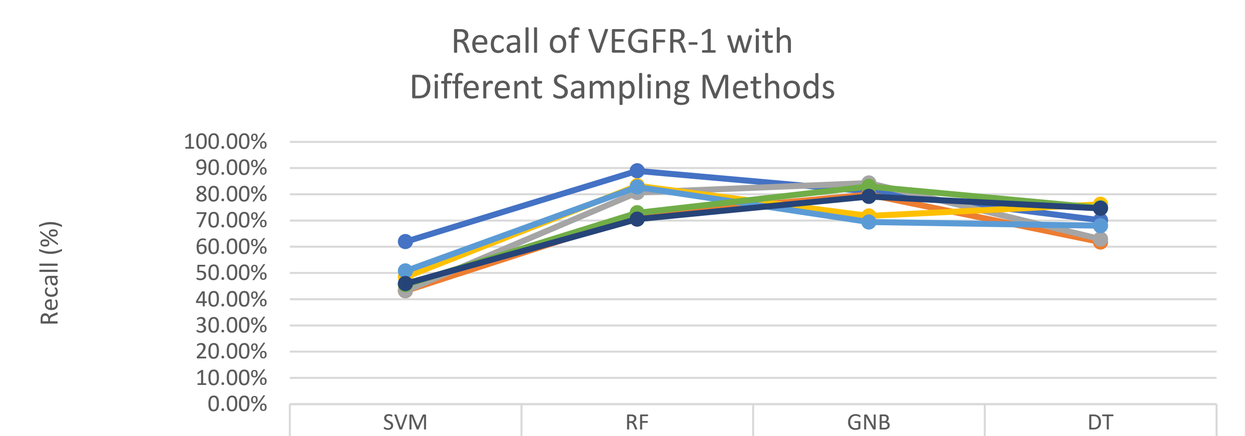

	SVM	RF	GNB	DT
None	61.97%	88.94%	81.06%	70.06%
SMOTE	43.19%	71.81%	79.90%	61.76%
RUS	43.19%	80.63%	84.27%	62.90%
ADASYN	48.44%	83.28%	71.74%	76.15%
BorderlineSMOTE	50.76%	82.78%	69.41%	68.01%
SVM-SMOTE	45.52%	72.91%	83.03%	74.81%
SMOTETomek	45.96%	70.50%	79.17%	74.64%

(c)

Figure S9. The accuracy, precision and recall values for VEGFR-1. **(a)** Accuracy of VEGFR-1 when different sampling methods are applied; **(b)** Precision of VEGFR-1 when different sampling methods are applied; **(c)** Recall of VEGFR-1 when different sampling methods are applied.

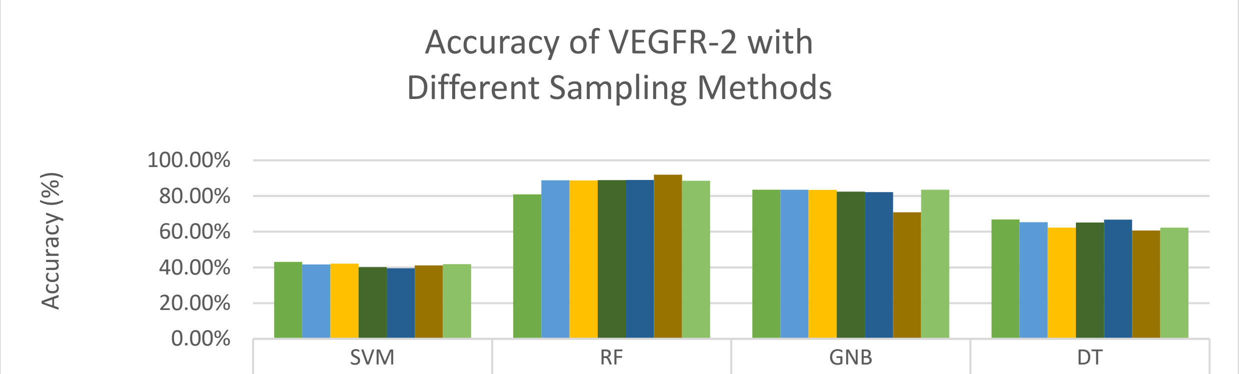

	SVM	RF	GNB	DT
None	43.07%	80.95%	83.51%	66.82%
SMOTE	41.68%	88.82%	83.54%	65.29%
RUS	42.07%	88.69%	83.45%	62.24%
ADASYN	40.17%	88.87%	82.45%	65.17%
BorderlineSMOTE	39.51%	88.99%	82.19%	66.79%
SVM-SMOTE	41.15%	91.94%	70.84%	60.65%
SMOTETomek	41.86%	88.54%	83.54%	62.27%

(a)

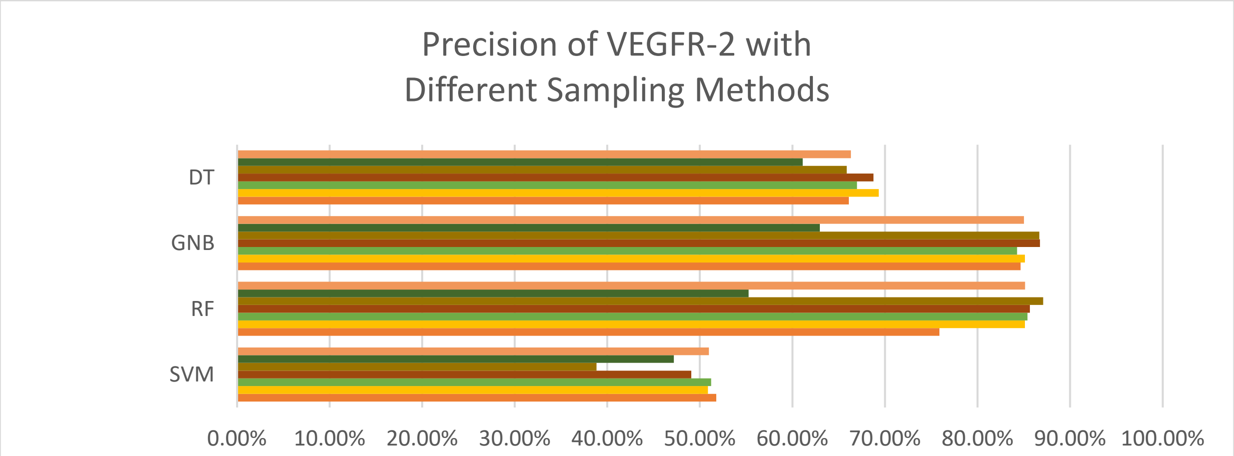

	SVM	RF	GNB	DT
SMOTETomek	50.98%	85.13%	85.02%	66.31%
SVM-SMOTE	47.19%	55.26%	62.97%	61.11%
BorderlineSMOTE	38.83%	87.08%	86.67%	65.87%
ADASYN	49.07%	85.65%	86.74%	68.75%
RUS	51.23%	85.39%	84.27%	66.98%
SMOTE	50.88%	85.11%	85.12%	69.33%
None	51.77%	75.88%	84.65%	66.09%

(b)

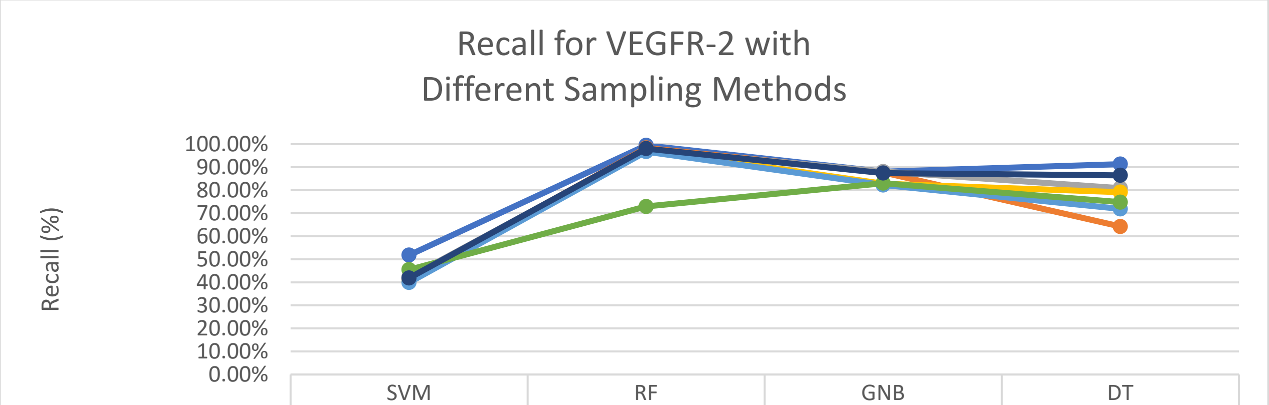

	SVM	RF	GNB	DT
None	51.82%	99.48%	88.03%	91.31%
SMOTE	41.40%	98.33%	87.65%	64.24%
RUS	42.26%	97.74%	88.09%	80.89%
ADASYN	40.30%	97.59%	82.81%	79.14%
BorderlineSMOTE	39.92%	96.76%	82.23%	71.87%
SVM-SMOTE	45.52%	72.91%	83.03%	74.81%
SMOTETomek	41.88%	98.09%	87.42%	86.45%

(c)

Figure S10. The accuracy, precision and recall values for VEGFR-2. **(a)** Accuracy of VEGFR-2 when different sampling methods are applied; **(b)** Precision of VEGFR-2 when different sampling methods are applied; **(c)** Recall of VEGFR-2 when different sampling methods are applied.

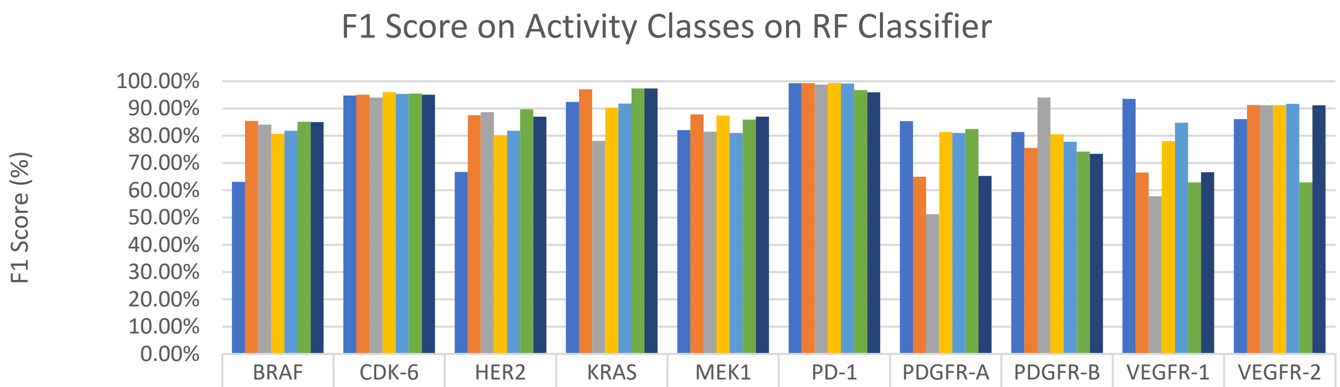

	BRAF	CDK-6	HER2	KRAS	MEK1	PD-1	PDGFR-A	PDGFR-B	VEGFR-1	VEGFR-2
None	63.07%	94.76%	66.70%	92.41%	82.05%	99.25%	85.37%	81.33%	93.50%	86.09%
SMOTE	85.41%	95.06%	87.49%	97.05%	87.83%	99.25%	64.94%	75.54%	66.51%	91.25%
RUS	84.02%	93.95%	88.60%	78.14%	81.47%	98.75%	51.22%	93.96%	57.81%	91.15%
ADASYN	80.67%	96.00%	80.05%	90.25%	87.38%	99.36%	81.37%	80.54%	78.00%	91.23%
BorderlineSMOTE	81.78%	95.34%	81.79%	91.79%	80.96%	99.14%	80.99%	77.76%	84.76%	91.67%
SVM-SMOTE	85.11%	95.47%	89.71%	97.34%	85.94%	96.76%	82.46%	74.15%	62.87%	62.87%
SMOTETomek	84.97%	95.06%	86.97%	97.32%	86.98%	95.90%	65.24%	73.36%	66.58%	91.15%

Figure S11. F1 scores for all activity classes with the RF classifier

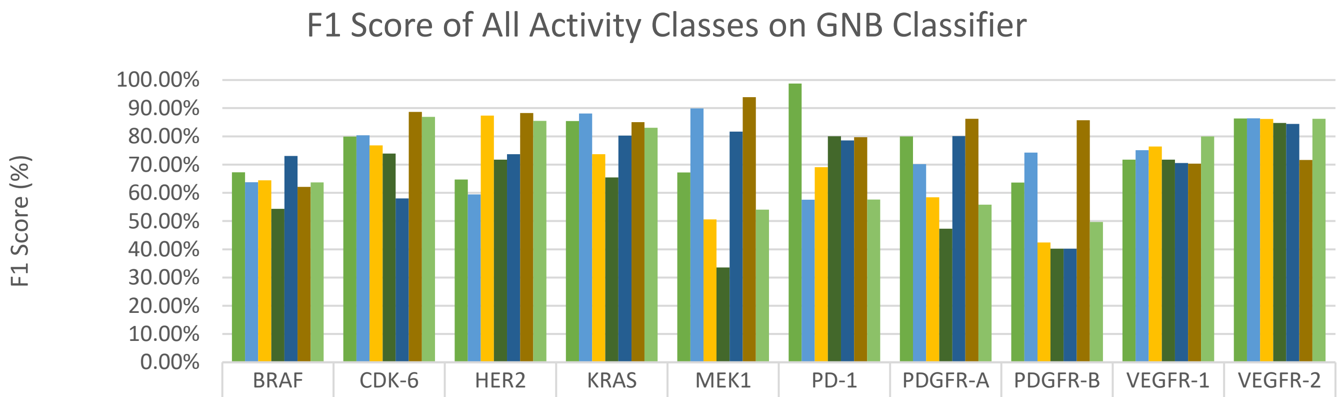

	BRAF	CDK-6	HER2	KRAS	MEK1	PD-1	PDGFR-A	PDGFR-B	VEGFR-1	VEGFR-2
None	67.24%	79.94%	64.73%	85.41%	67.20%	98.71%	79.96%	63.64%	71.72%	86.31%
SMOTE	63.75%	80.39%	59.44%	88.11%	89.88%	57.55%	70.17%	74.22%	75.07%	86.37%
RUS	64.41%	76.82%	87.36%	73.66%	50.59%	69.06%	58.40%	42.42%	76.39%	86.14%
ADASYN	54.33%	73.92%	71.73%	65.46%	33.56%	80.04%	47.29%	40.22%	71.74%	84.73%
BorderlineSMOTE	73.04%	58.04%	73.65%	80.26%	81.66%	78.55%	80.07%	40.20%	70.56%	84.39%
SVM-SMOTE	62.12%	88.68%	88.28%	85.02%	93.87%	79.67%	86.19%	85.68%	70.31%	71.62%
SMOTETomek	63.68%	86.90%	85.51%	83.02%	54.05%	57.63%	55.78%	49.65%	80.00%	86.20%

Figure S12. F1 score for all activity classes with the GNB classifier
